# Function of KCNQ2 channels at nodes of Ranvier of lumbar spinal ventral nerves of rats

**DOI:** 10.1186/s13041-022-00949-0

**Published:** 2022-07-20

**Authors:** Sotatsu Tonomura, Jennifer Ling, Jianguo G. Gu

**Affiliations:** Department of Anesthesiology and Perioperative Medicine, School of Medicine, University of Alabama at Birmingham, Birmingham, AL 35294 USA

**Keywords:** Node of Ranvier, Voltage-gated K^+^ channel, KCNQ2 channel, Kv7.2 channels, Action potential, Saltatory conduction, Motor nerves

## Abstract

Previous immunohistochemical studies have shown the expression of KCNQ2 channels at nodes of Ranvier (NRs) of myelinated nerves. However, functions of these channels at NRs remain elusive. In the present study, we addressed this issue by directly applying whole-cell patch-clamp recordings at NRs of rat lumbar spinal ventral nerves in ex vivo preparations. We show that depolarizing voltages evoke large non-inactivating outward currents at NRs, which are partially inhibited by KCNQ channel blocker linopirdine and potentiated by KCNQ channel activator retigabine. Furthermore, linopirdine significantly alters intrinsic electrophysiological properties of NRs to depolarize resting membrane potential, increase input resistance, prolong AP width, reduce AP threshold, and decrease AP amplitude. On the other hand, retigabine significantly decreases input resistance and increases AP rheobase at NRs. Moreover, linopirdine increases excitability at NRs by converting single AP firing into multiple AP firing at many NRs. Saltatory conduction velocity is significantly reduced by retigabine, and AP success rate at high stimulation frequency is significantly increased by linopirdine. Collectively, KCNQ2 channels play a significant role in regulating intrinsic electrophysiological properties and saltatory conduction at NRs of motor nerve fibers of rats. These findings may provide insights into how the loss-of-function mutation in KCNQ2 channels can lead to neuromuscular disorders in human patients.

## Introduction

The propagation of action potentials (AP) through nodes of Ranvier (NRs) [21, 22] along myelinated nerves, known as saltatory conduction [4, 13, 19, 27], is essential for neuronal function, and has important implications in neurological diseases [2, 11, 26, 30, 31]. The NRs of mammalian myelinated nerves use two-pore domain K^+^ channels (K2P) as principal K^+^ channels [15], which is critical in AP regeneration as well as high-speed and high-frequency saltatory conduction at NRs [15, 17]. Although immunohistochemical studies have shown the expression of voltage-gated K^+^ channel KCNQ2 at NRs of both peripheral and central nerves [10, 25], their functional role at these sites remains elusive. Voltage-gated K^+^ channels appear to be functionally insignificant in trigeminal Aβ-afferent nerves of rats as reported in our previous study [15]. However, in lumbar spinal ventral nerves, we have recently shown that voltage-gated K^+^ channel blockers TEA and 4-AP plus Cs^+^ alter both passive and active membrane properties at NRs, suggesting a significant role of voltage-gated K^+^ channels in regulating intrinsic electrophysiological properties [28]. It is currently unknown whether KCNQ2 channels are functional voltage-gated K^+^ channels at NRs of lumbar spinal ventral nerves and if so, what specific roles KCNQ2 channels may play in intrinsic electrophysiological properties and saltatory conduction at NRs of the motor nerves.

KCNQ2 belongs to KCNQ subfamily (KCNQ1 to KCNQ5) of voltage-gated K^+^ channels [7, 14, 23]. KCNQ channels are activated at a low threshold near resting membrane potential, and do not become inactivated, thereby contributing to the setting of the resting membrane potential and regulation of neuronal excitability [7, 14]. KCNQ channels, especially KCNQ2 and KCNQ3, mediate a slow and non-inactivating M-type outward K^+^ current (M-current), which is inhibited when muscarinic receptors are activated [6, 7, 29]. KCNQ channels can also be directly inhibited by compounds such as linopirdine and XE991, as well as potentiated by compounds such as retigabine [1, 7, 33]. Many neurons in the peripheral nervous system (PNS) and central nervous system (CNS) express KCNQ2, KCNQ3, and KCNQ5 channels [7]. KCNQ2 and KCNQ3 are found to be co-localized in several neuronal populations and may form heteromeric channels [14, 29]. KCNQ2 channels appear to be expressed at all NRs of PNS nerves, including the sciatic nerve, the dorsal and ventral roots, and intramuscular nerves [10]. KCNQ2 and KCNQ3 channels are found to be co-expressed in small- and intermediate-diameter myelinated peripheral nerves [25]. However, at NRs of large-diameter axons of rat peripheral nerves, KCNQ2 channels but not KCNQ3, are expressed at NRs, and homomeric KCNQ2 are thought to be the functional channels at NRs of these nerves [10, 25].

Loss-of-function mutations in KCNQ2 and KCNQ3 channels occur in a small human population, causing a number of neurological disorders such as myokymia, neuromyotonia, neonatal-onset epilepsy, and epileptic encephalopathy [8, 14, 18, 20, 32]. It has been reported that a KCNQ2 mutation (R207W) causes myokymia in human patients [8]. Myokymia is a neuromuscular disorder in human patients manifested with involuntary, repetitive muscle contraction [12]. In myokymia, APs are repetitively generated at myelinated motor axons, resulting in repetitive activity of motor units [12]. Neuromuscular disorders associated with KCNQ2 loss-of-function mutations strongly suggest that KCNQ2 channels have important functions at NRs of motor nerve fibers. However, the degree to which KCNQ2 channels account for functional K^+^ channels at NRs of motor nerves has not been quantitatively investigated. Furthermore, the specific roles of KCNQ2 channels in regulating intrinsic electrophysiological properties and saltatory conduction at NRs of motor nerve fibers remain unclear. In the present study, we aimed to address these questions by using ex vivo lumbar spinal ventral nerve preparation and whole-cell patch-clamp recordings at NRs.

## Materials and methods

### Ex vivo lumbar spinal ventral nerve preparation

Sprague Dawley rats of both males and females aged at 10–14 weeks were deeply anesthetized with isoflurane and then sacrificed by decapitation. L4 and L5 lumbar spinal ventral nerves (~ 15 mm) were dissected out and placed in a petri dish filled with a Krebs solution containing (in mM): 117 NaCl, 3.5 KCl, 2.5 CaCl_2_, 1.2 MgCl_2_, 1.2 NaH_2_PO_4_, 25 NaHCO_3_, and 11 glucose. The Krebs solution was saturated with 95% O_2_ and 5% CO_2_, and had pH of 7.35 and osmolarity of 324 mOsm. Connective tissues on the surface of the nerve bundles were removed with a pair of fine forceps under a dissection microscope, and were then affixed in a recording chamber using a tissue anchor, and submerged in the Krebs solution. The recording chamber was mounted on the stage of an Olympus BX51 microscope that was equipped with IR-DIC and fluorescent imaging systems. To facilitate penetration of the patch-clamp recording electrode through perineural tissues, lumbar spinal ventral nerves were briefly exposed to a mixture of 0.07% dispase II (Roche, Indianapolis, IN, USA) and 0.07% collagenase (MilliporeSigma, Billerica, MA, USA) in Krebs solution for 5–10 min, and the enzymes were then washed off with Krebs solution. The ex vivo nerve preparation was continuously perfused with the Krebs solution at 24 °C maintained by a Peltier temperature control system (CL-200A, Warner Instrument, CT, USA).

### Pressure-patch-clamp recordings at nodes of Ranvier

Nodes of Ranvier in lumbar spinal ventral nerves were visualized under a 40x (NA 0.80) water immersion objective with images captured by an infrared CCD camera (IR-1000, DAGE-MTI, USA). Patch-clamp recordings were performed at NRs of lumbar spinal ventral nerves. Recording electrodes were pulled with a Flaming/Brown Micropipette Puller (P-97, Shutter Instruments, CA, USA). Electrode resistance after filling recording electrode internal solution ranged from 5 to 8 MΩ for patch-clamp recordings at NRs. Recording electrodes were filled with a K^+^-based internal solution containing (in mM): 105 K-gluconate, 30 KCl, 0.5 CaCl_2_, 2.4 MgCl_2_, 5 EGTA, 10 HEPES, 5 Na_2_ATP and 0.33 GTP-TRIS salt; the pH of the solution was adjusted to 7.35 with KOH. To access axonal membranes at NRs by recording electrodes and achieve high quality membrane seals, a high-speed pressure-clamp device (HSPC-1, ALA Scientific instruments, NY, USA) was connected to the electrode holder to fine control internal pressures of the patch-clamp recording electrode while approaching nodal membranes. The pressure-patch-clamp recordings were performed in the same manner as described in our previous studies [15, 16]. Signals of voltage-clamp experiments were recorded and amplified using a multiclamp 700B amplifier, filtered at 10 kHz and sampled at 10 kHz using the pCLAMP 11 software (Molecular Devices, Sunnyvale, CA, USA). Signals of current-clamp recordings for action potentials at NRs were low-pass filtered at 10 kHz and sampled at 50 kHz.

To measure ionic currents flowing through axonal membranes at NRs following voltage steps, recordings were performed under whole-cell voltage-clamp configuration with nodal membranes held at − 72 mV. Voltage steps were applied from − 112 mV to + 58 mV (voltage command of − 100 to + 70 mV) with increments of 10 mV each step and a step duration of 500 ms. Unless otherwise indicated, membrane voltages mentioned in the texts have been corrected for the calculated junction potentials of 12.3 mV. To determine the properties of membranes and action potentials at NRs, patch-clamp recordings were performed under whole-cell current-clamp configuration. Step current pulses were injected into NRs through recording electrodes. Step currents were applied from − 100 pA to 2900 pA with increments of 50 pA per step and the duration of each pulse was 50 ms.

### Determination of saltatory conduction velocity and success rate along lumbar spinal ventral nerves

To determine conduction velocity of APs through NRs on a lumbar spinal ventral nerve, APs were orthodromically evoked at a distal site of the nerve bundle using a suction stimulation electrode. The distance between the stimulation site and recording site was approximately 15 mm. The suction stimulation electrode tip size was approximately 0.5 mm in diameter and fire-polished. The distal nerve end was aspirated into the suction stimulation electrode to produce a tight fitting by a negative pressure. The negative pressure was continuously applied to the suction stimulation electrode to maintain tight fitting during experiments. To initiate APs at the distal end of the nerve bundle, monophasic square wave pulses were generated by an electronic stimulator (Master-8, A.M.P.I, Israel) and delivered via a stimulation isolator (ISO-Flex, A.M.P.I, Israel) to the suction stimulation electrode. The duration of the stimulation pulse was 50 µs. Minimum stimulation intensity for evoking APs, i.e., stimulation threshold, was first determined. Then stimulation was applied at the intensity of twofold threshold throughout the experiments. Conduction velocity was calculated based on the latency of APs and the axonal length. The latency of APs was measured from the time of stimulation that was marked by stimulation artifacts to the time when AP was initiated at recording site. Axonal length was measured as the distance between stimulation and recording sites. To determine success rate of APs at NRs following different stimulation frequencies, stimulation pulses were applied to nerve bundles at frequencies of 1, 10, 50, 100, 200, 500 and 1000 Hz. Stimulation at each frequency was applied for 20 s. Intervals between tests at different stimulation frequencies were 30 s. Success rate of APs conducted through NRs was the percentage of successfully propagated APs through the recording site during a 20-s period of stimulation.

### Pharmacology

Effects on intrinsic electrophysiological properties at NRs by KCNQ2 channel blocker linopirdine and activator retigabine were examined. In experiments, recordings were performed in the absence (control) and presence of 100 μM linopirdine (Tocris Bioscience) or 10 μM retigabine (Adooq Bioscience). Testing compounds were bath applied, and each compound was perfused to ex vivo nerve preparations for 10 min.

### Immunohistochemistry

Rats of both males and females aged at 10–14 weeks were deeply anesthetized with isoflurane and then sacrificed by decapitation. L4 and L5 lumbar spinal ventral nerves (~ 15 mm) were dissected out and rapidly frozen in powdered dry ice. The lumbar spinal ventral nerves were sectioned longitudinally at the thickness of 10 µm each section at − 21 °C using a Leica CM1860 cryomicrotome. Sections were air dried for 6 min and fixed with − 20 °C acetone for 7 min and air dried for an additional 30 min. The sections were washed in PBS 3 times each for 10 min. They were incubated in a blocking solution of 10% normal goat serum in PBS for 30 min at room temperature. They were then incubated with primary antibodies in dilution buffer (5% normal goat serum in PBS) overnight at 4 °C. After washing the sections in PBS 3 times each 10 min, the sections were incubated with a secondary antibody in dilution buffer (5% normal goat serum in PBS) for 1 h at room temperature. Following 3 washes in PBS each for 10 min, anti-fade mountant (ProLong Diamond Antifade Mountant, Invitrogen, Carlsbad, CA, USA) was added and sections were covered by cover-glasses. Following primary antibodies were used: rabbit anti-KCNQ2 (1:500, Alomone labs, Jerusalem, Israel), and mouse anti-contactin associated protein 1 (CASPR, 1:500, MilliporeSigma, Burlington, MA, USA). The following secondary antibodies were used for visualizing the immunohistochemistry results. They were Alexa Fluor 594-conjugated goat anti-rabbit (1:1000, Invitrogen, Carlsbad, CA, USA), and Alexa Fluor 488-conjugated goat anti-mouse (1:1000, Invitrogen, Carlsbad, CA, USA).

### Data analysis

Electrophysiological data were analyzed using Clampfit 11 (Molecular Devices). Data collected from lumbar spinal ventral nerves of 3 male and 7 female animals were aggregated for data analysis since no differences in electrophysiological results were found between male and female animals. Input resistance was determined with a − 10 mV voltage step from the membrane holding voltage of − 72 mV. Resting membrane potentials (RMPs) were measured under current-clamp configuration at zero holding current. AP rheobase was the threshold step current that evoked AP firing. AP threshold was the threshold potential at which AP upstroke started. AP amplitude was measured from RMP to AP peak. AP width was measured as the duration from 50% AP upstroke to 50% AP repolarization. All the above AP parameters were determined with the AP evoked by the rheobase step current at NRs. AP success rate at NRs was calculated as the number of APs recorded at NRs divided by the number of electrical stimuli, where the electrical stimuli were applied to the lumbar spinal ventral nerves at frequencies for 20 s. All data analyses were performed using Graph Pad Prism (version 8). Unless otherwise indicated, all data were reported as mean ± SEM of *n* independent observations. Statistical significance was evaluated using paired Student’s t test or Chi-square test. Differences were considered to be significant with *p < 0.05, **p < 0.01, ***p < 0.001, and not significant (ns) with p ≥ 0.05.

## Results

### Linopirdine-sensitive K^+^ currents and KCNQ2 expression at nodes of Ranvier of lumbar spinal ventral nerves

**Fig. 1:**
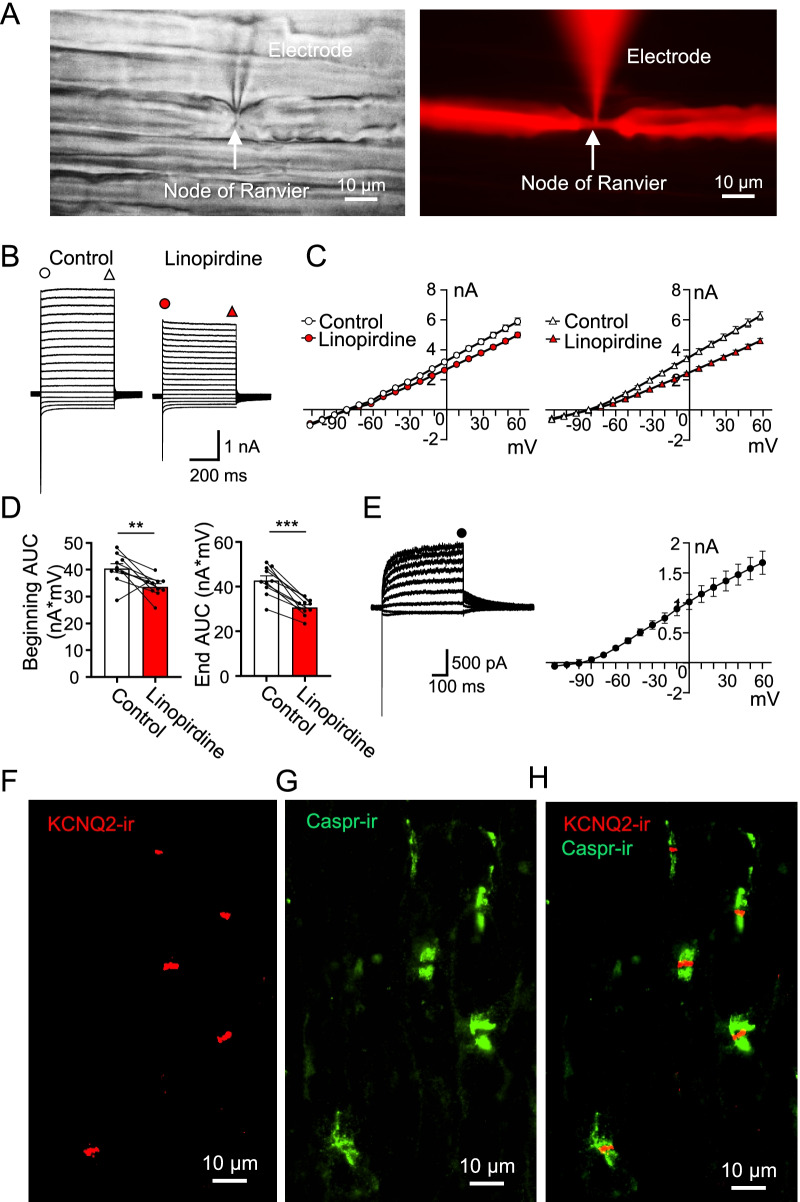
Linopirdine-sensitive non-inactivating K^+^ currents and KCNQ2 expression at NRs of lumbar spinal ventral nerves. **A** Bright image (left) and fluorescent image (right) show the same region of an L5 lumbar spinal ventral nerve viewed under a 40 × objective. Arrow indicates a node of Ranvier (NR) and a patch-clamp recording electrode tip. Alexa Fluor 555 (85 μM) was included in the recording electrode internal solution for axon labelling. **B** Sample traces show currents recorded at a NR following voltage steps in the absence (left, control) and presence of 100 µM linopirdine (right). **C** I–V curves at the beginning part (left panel) and the end part (right panel) of non-inactivating outward currents in the control (n = 10, open symbols) and the presence of linopirdine (n = 10, red symbols). **D** Area under the I–V curve (AUC) of the beginning (left panel) and the end part (right panel) of non-inactivating outward currents in the control (n = 10, open bar) and the presence of linopirdine (n = 10, red bar). **E** Left panel, sample traces show linopirdine-sensitive outward currents obtained by subtraction of total outward currents in the presence of linopirdine from total currents in the control. Right panel, I–V curves of the linopirdine-sensitive outward currents at the end part (n = 10, solid circles). **F**, **G** KCNQ2-immunoreactivity (KCNQ2-ir, **F**) and caspr-ir (**G**) on lumbar spinal ventral nerves. **H** Overlay image of (**F**) and (**G**) shows that KCNQ2-ir is located in the space between caspr-ir, the region of NRs. Data represent mean ± SEM, **p < 0.01, ***p < 0.001, paired Student’s t-Test

We performed whole-cell patch-clamp recordings to investigate whether KCNQ2 may mediate outward K^+^ currents at NRs of lumbar spinal ventral nerves of rats. Figure 1A is an example showing a recording made at a NR of a lumbar spinal ventral nerve. In this recording, the fluorescent dye Alexa Fluor 555 was included in recording electrode internal solution to label axons. The nodal axon (narrow part) and internodal axon could be visualized under a fluorescent microscope (Fig. 1A right). Under voltage-clamp configuration, we examined membrane currents following the application of depolarizing voltage steps at NRs. Depolarizing voltage steps evoked large transient inward currents which were immediately followed by large non-inactivating outward currents (Fig. 1B). The non-inactivating outward currents were partially inhibited by 100 µM linopirdine, a potent blocker of KCNQ channels (Fig. 1B, C). Figure 1C shows current–voltage relationship (I–V curve) of the non-inactivating outward currents at the beginning (Fig. 1C left) and the end (Fig. 1C right) of the voltage steps. Both the beginning and the end parts of the non-inactivating outward currents were reduced following the application of 100 µM linopirdine. For example, at the voltage step to 18 mV, the non-inactivating outward currents at the beginning were 4.1 ± 0.2 nA (n = 10) in the control, and significantly reduced to 3.4 ± 0.1 nA (n = 10) in the presence of linopirdine (p < 0.001, t = 3.4, Fig. 1C left). The non-inactivating outward currents at the end part were 4.4 ± 0.2 nA in the control, and significantly reduced to 3.1 ± 0.1 nA (n = 10) in the presence of linopirdine (p < 0.001, t = 7.8, Fig. 1C right). Expressed as the area under the I–V curve (AUC), non-inactivating outward currents at the beginning were 40.5 ± 1.7 nA*mV in the control, and significantly reduced to 33.6 ± 1.2 nA*mV in the presence of linopirdine (n = 10, P < 0.01, t = 7.9, Fig. 1D left). The non-inactivating outward currents at the end were 42.7 ± 2.0 nA*mV in the control, and significantly reduced to 30.6 ± 1.1 nA*mV in the presence of linopirdine (n = 10, P < 0.001, t = 3.5, Fig. 1D right). Linopirdine-sensitive non-inactivating currents were isolated after subtraction of the total currents in the presence of 100 μM linopirdine from the total currents in the absence of linopirdine (Fig. 1E left). The isolated outward currents displayed slow activation and inactivation with tail currents (Fig. 1E left). Reversal potentials of the isolated outward currents were − 82.6 ± 0.9 mV (n = 10, Fig. 1E right). These results indicate that a small but significant fraction of non-inactivating outward current at NRs is mediated by linopirdine-sensitive KCNQ channels. Consistent with the above electrophysiological and pharmacological results, immunohistochemical study with anti-KCNQ2 antibody showed strong KCNQ2-immunoreactivity (KCNQ2-ir) at NRs of lumbar spinal ventral nerve fibers (Fig. 1F–H). KCNQ2-ir was sandwiched by caspr-immunoreactivity (caspr-ir), which outlined paranodal regions on myelinated nerves (Fig. 1H).

**Fig. 2:**
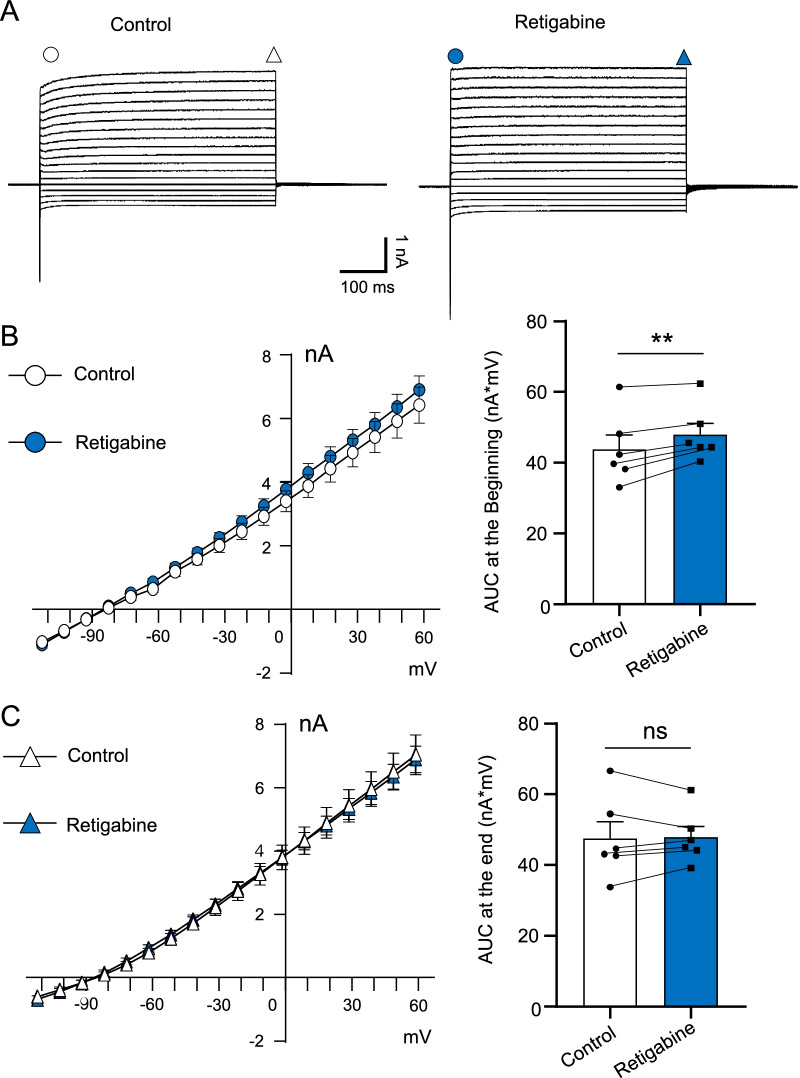
Effects of retigabine on non-inactivating outward currents at NRs. **A** Two sets of sample traces show currents recorded at a NR following voltage steps in the absence (left, control) and presence of 10 µM retigabine (right). Circles and triangles indicate the beginning part and the end part of the outward currents, respectively. Currents at these two parts are used to plot I–V relationship in (**B**) and (**C**). **B** Left panel, I–V curve of the beginning part of the outward currents in the control (n = 6, open circle) and the presence of retigabine (n = 6, blue circle). Right panel, area under the I–V curve (AUC) of the beginning part of the outward currents in the control (n = 6, open bar) and the presence of retigabine (n = 6, blue bar). **C** Left panel, I–V curve of the end part of the outward currents in the control (n = 6, open triangle) and the presence of retigabine (n = 6, blue triangle). Right panel, AUC of the end part of the outward currents in the control (n = 6, open bar) and the presence of retigabine (n = 6, blue bar). AUC was used for statistical comparison of non-inactivating outward currents in the control and the presence of retigabine. Data represent mean ± SEM, ns, not significantly different, **p < 0.01, paired Student’s t-Test

We next measured the effects of KCNQ channel activator retigabine on non-inactivating outward currents at NRs. At the beginning components, non-inactivating outward currents were slightly increased following the application of 10 µM retigabine (n = 6, Fig. 2A, B). AUC at the beginning part of the outward currents was 43.8 ± 4.7 nA*mV (n = 6) in the control, and significantly increased to 48.0 ± 3.2 nA*mV (n = 6, p < 0.01, t = 4.4) in the presence of retigabine (Fig. 2B right). However, we did not observe any significant difference in the end part of the non-inactivating outward currents between the control and in the presence of retigabine (Fig. 2C). Therefore, KCNQ2 channels expressed at NRs of lumbar spinal ventral nerves partially mediate non-inactivating outward K^+^ currents with sensitivity to linopirdine and retigabine observed.

### Role of KCNQ2 channels in regulating intrinsic electrophysiological properties at nodes of Ranvier

**Fig. 3:**
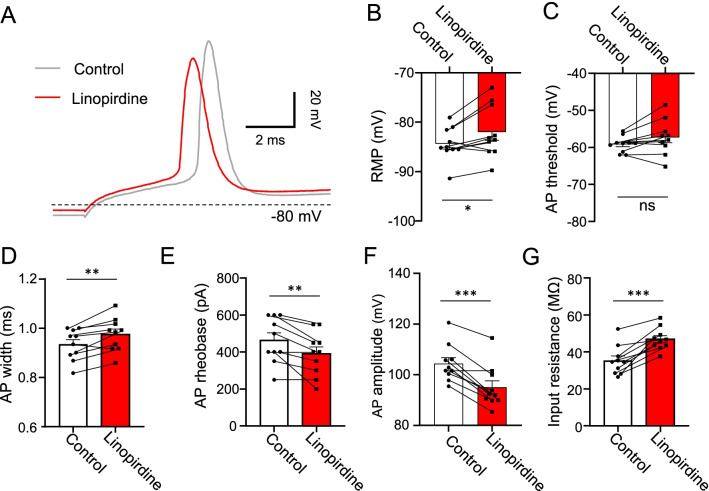
Effects of linopirdine on intrinsic electrophysiological properties at NRs. **A** Sample traces illustrate action potentials (APs) recorded at a NR in the absence (control, grey) and presence of 100 µM linopirdine (red). Recordings were performed under the whole-cell current-clamp configuration and step currents were injected into the NR through recording electrodes. **B**–**G** Bar graphs show, in the absence (control, open bars, n = 10) and presence of linopirdine (red bars, n = 10), intrinsic electrophysiological properties including resting membrane potential (RMP, **B**), action potential (AP) threshold (**C**), AP width (**D**), AP rheobase (**E**), AP amplitude (**F**), and membrane input resistance (**G**). Data represent mean ± SEM, ns, not significantly different, *p < 0.05, **p < 0.01, ***p < 0.001, paired Student’s t-Test

To determine potential role of KCNQ2 channels in regulating intrinsic electrophysiological properties of nodal membranes, we investigated whether inhibition of KCNQ2 channels by linopirdine (100 µM) could significantly alter membrane and action potential properties at NRs of the lumbar spinal ventral nerves. Under current-clamp configuration, we measured resting membrane potential (RMP) at NRs, then depolarized NRs from their RMP by injecting depolarizing current steps to evoke APs at NRs (Fig. 3A). RMP at NRs was − 84.4 ± 1.1 mV (n = 10) in the absence of linopirdine (control), and significantly depolarized to − 82.0 ± 1.7 mV (n = 10) in the presence of linopirdine (Fig. 3B, p < 0.05, t = 2.9). AP threshold was − 59.5 ± 0.6 mV (n = 10) in the control group, and − 57.4 ± 1.5 mV (n = 10) in the presence of linopirdine, and was not significantly different (Fig. 3C). AP width was 0.88 ± 0.02 ms (n = 10) in the control group, and significantly prolonged to 0.93 ± 0.03 ms (n = 10) in the presence of linopirdine (Fig. 3D, p < 0.01, t = 3.8). AP rheobase was 465.0 ± 38.8 pA (n = 10) in the control group, and significantly decreased to 390.0 ± 37.1 pA (n = 10) in the presence of linopirdine (Fig. 3E, p < 0.01, t = 3.7). AP amplitude was 104.4 ± 2.3 mV (n = 10) in the control group, and significantly decreased to 95.0 ± 2.6 mV (n = 10) in the presence of linopirdine (Fig. 3F, p < 0.001, t = 4.9). Input resistance, measured under the voltage-clamp configuration following a − 10 mV testing pulse from membrane holding voltage at − 72 mV, was 35.3 ± 2.5 MΩ (n = 10) in the control group, and significantly increased to 46.9 ± 1.9 MΩ (n = 10) in the presence of linopirdine (Fig. 3G, p < 0.001, t = 6.7).

**Fig. 4:**
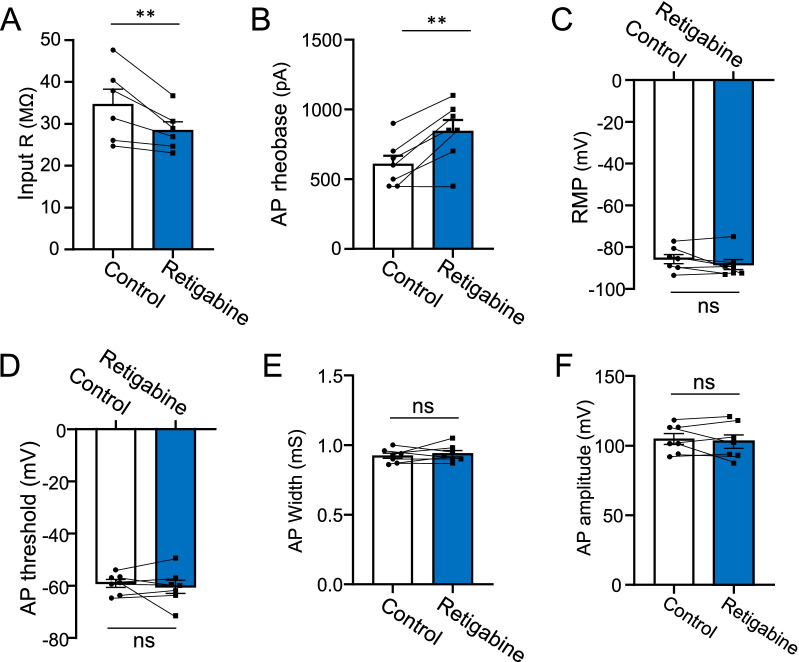
Effects of retigabine on intrinsic electrophysiological properties at nodes of Ranvier. **A**–**F** Bar graph shows, in the absence (control, open bars) and presence of 10 µM retigabine (Blue bars), intrinsic electrophysiological properties at NRs including membrane input resistance (**A,** n = 6), AP rheobase (**B,** n = 7), RMP (**C**, n = 7), AP threshold (**D**, n = 7), AP width (**E**, n = 7), and AP amplitude (**F**, n = 7). Data represent mean ± SEM, ns, not significantly different, **p < 0.01, paired Student’s t-Test

We determined effects of retigabine (10 µM) on intrinsic electrophysiological properties at NRs (Fig. 4). Input resistance was 34.7 ± 3.6 MΩ (n = 6) in the control group, and significantly decreased to 28.5 ± 2.0 MΩ (n = 6) in the presence of retigabine (Fig. 4A, p < 0.01, t = 3.4). AP rheobase was 607.1 ± 61.2 pA (n = 7) in the control group, and significantly increased to 842.9 ± 81.2 pA (n = 7) in the presence of retigabine (Fig. 4B, p < 0.01, t = 4.7). Other membrane and AP properties including RMP, AP threshold, AP width and AP amplitude were not significantly different between the control group and in the presence of 10 µM retigabine (Fig. 4C–F).

### Role of KCNQ2 channels in controlling action potential firing patterns at nodes of Ranvier

**Fig. 5:**
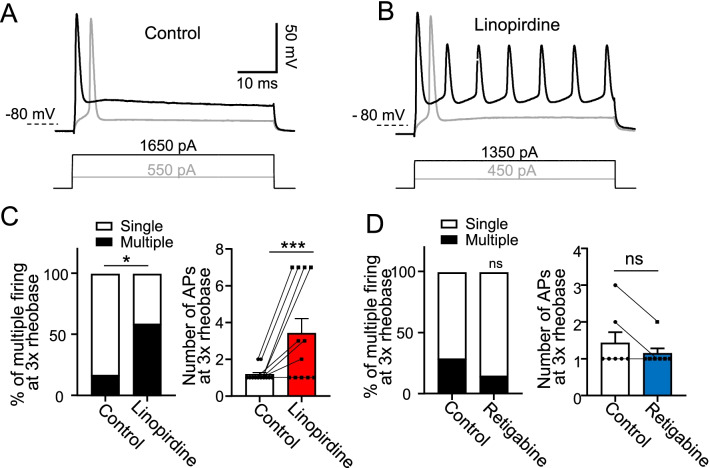
Effects of linopirdine and retigabine on AP firing patterns at nodes of Ranvier. **A**, **B** Sample traces show APs evoked at a NR by current steps in the absence (control, **A**) and presence of 100 µM linopirdine (**B**). The grey and black traces in (**A**) and (**B**) show APs evoked with rheobase (grey) and 3 × rheobase (black) stimulation, respectively. Recordings were performed under the whole-cell current-clamp configuration and step currents were injected into the NR through recording electrodes. **C** Left panel, percent of NRs with single AP firing (open bar) and multiple AP firing (black bar) before (control, n = 12) and following the application of linopirdine (n = 12). Right panel, Numbers of APs before (control, n = 12) and following the application of linopirdine (n = 12). **D** Similar to C except retigabine was tested (n = 7). Data represent mean ± SEM, ns, not significantly different, *p < 0.05, ***p < 0.001, paired Student’s t-Test

At NRs a supra-threshold depolarizing current step usually only evoked a single AP (Fig. 5A, C). We determined whether KCNQ2 channels play a role in the AP firing pattern at NRs. This was done by investigation of AP firing patterns at NRs following the inhibition of KCNQ2 channels by linopirdine (Fig. 5B, C). In this set of experiments, APs were evoked by step currents at 3 × rheobase for 50 ms in the absence (control) and presence of 100 µM linopirdine. As shown in Fig. 5A–C, the percent of NRs showing single AP firing was 83.3% and multiple AP firing was 16.7% in the control group (n = 12, Fig. 5C left). The NRs with single AP firing significantly reduced to 41.7% and multiple AP firing significantly increased to 58.3% (n = 12) in the presence of linopirdine (Fig. 5C left, p < 0.05). Averaged AP numbers were analyzed, which were 1.2 ± 0.1 (n = 12) in the control group, and significantly increased to 3.4 ± 0.8 (n = 12) in the presence of 100 µM linopirdine (Fig. 5C right, p < 0.001, t = 2.4). We also tested effects of retigabine (10 µM) on AP firing patterns at NRs. The percent of NRs showing single AP firing was 71.5% and multiple AP firing was 28.5% (n = 7) in the control group (Fig. 5D left). The NRs showing single AP firing was 85.8% and multiple AP firing was 14.2% (n = 7) in the presence of 10 µM retigabine (Fig. 5D left), and were not significantly different from the control group. Averaged AP numbers were 1.4 ± 0.3 (n = 7) in the control group, and 1.1 ± 0.1 (n = 7) in the presence of 10 µM retigabine (Fig. 5D right), and were not significantly different.

### Role of KCNQ2 channels in regulating saltatory conduction

**Fig. 6:**
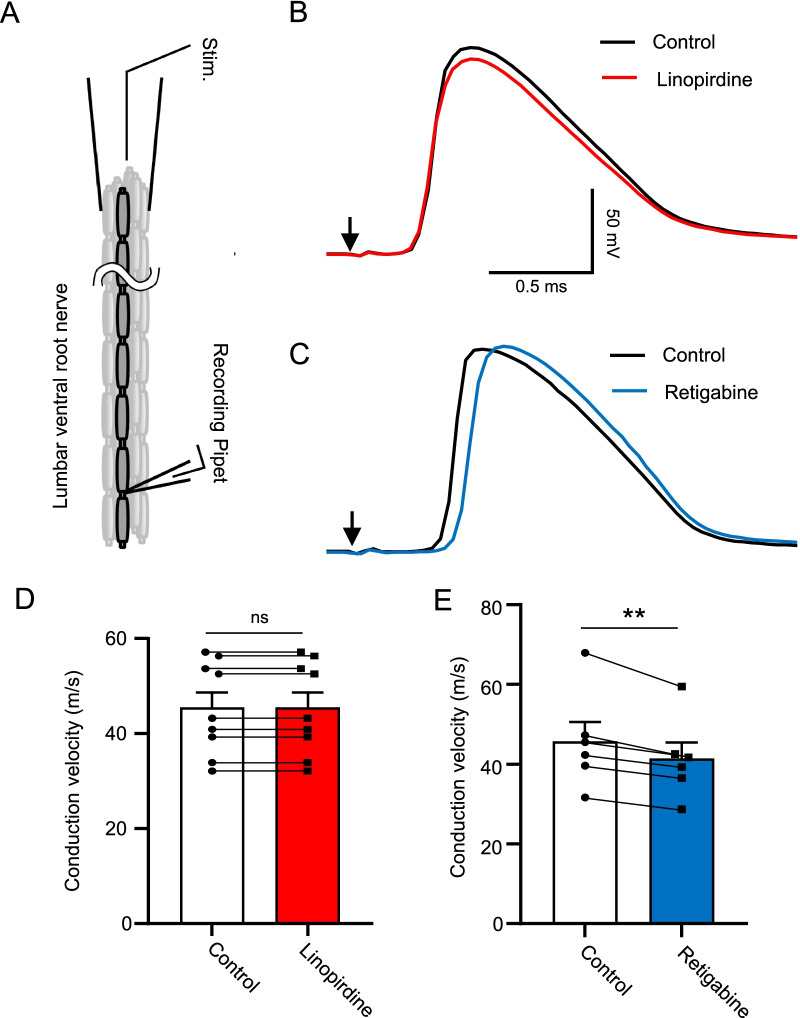
Effects of linopirdine and retigabine on saltatory conduction velocity. **A** Experimental setting for recording of AP propagation through NRs of lumbar spinal ventral nerves. APs were evoked from the distal site of the nerve bundle. **B** Two overlay sample traces show APs recorded from a NR following electrical stimulation. The recordings were performed in the absence (control, black) and presence of 100 µM linopirdine (red). **C** Two overlay sample traces show APs recorded from a NR following electrical stimulation. The recordings were performed in the absence (control, black) and presence of 10 µM retigabine (blue). In both (**B**) and (**C**), arrow indicates stimulation artifact. **D** Summary data of AP conduction velocity determined from the recordings exemplified in (**B**) in the control (n = 9) and the presence of linopirdine (n = 9). **E** Summary data of AP conduction velocity determined from the recordings exemplified in (**C**) in the control (n = 6) and the presence of retigabine (n = 6). Data represent mean ± SEM, ns, not significantly different, **p < 0.01, paired Student’s t-Test

We investigated whether KCNQ2 channels at NRs may play a role in regulating saltatory conduction velocity. In this set of experiments, we performed whole-cell patch-clamp recordings at NRs while APs were elicited by electrical stimulation applied to a distal site of lumbar spinal ventral nerves (Fig. 6A). Recordings were performed in the absence (control) and presence of 100 μM linopirdine (Fig. 6B), or in the absence and presence of 10 μM retigabine (Fig. 6C). AP conduction velocity was 45.4 ± 3.2 m/s (n = 9) in the absence of linopirdine, and not changed (45.4 ± 3.2 m/s, n = 9) in the presence of 100 μM linopirdine (Fig. 6D). AP conduction velocity was 45.7 ± 5.0 m/s (n = 6) in the control group, and significantly decreased to 41.3 ± 4.2 m/s (n = 6) in the presence of 10 µM retigabine (Fig. 6E, p < 0.01, t = 5.0).

**Fig. 7:**
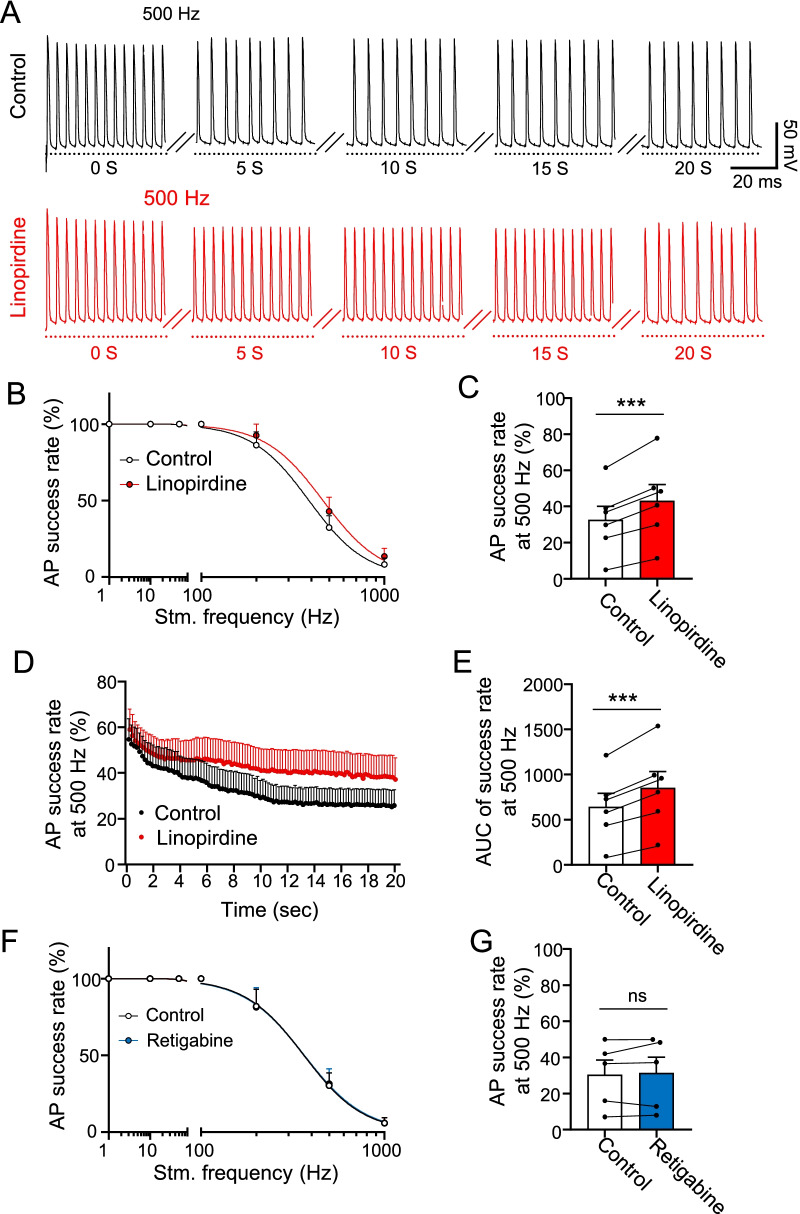
Effects of linopirdine and retigabine on high-frequency saltatory conduction. **A** Two sets of sample trances show APs recorded at a NR following a train of electrical stimulation at 500 Hz in control (top panel) and the presence of 100 µM linopirdine (bottom panel). Stimulation was applied for a period of 20 s. Only a short period of APs at initial time point (0 s), 5 s, 10 s, 15 s and 20 s time point are presented to illustrate changes of AP success rates over the time period of 20 s. **B** AP success rate at NRs with distal stimulation at frequency of 1, 10, 100, 200, 500 and 1000 Hz in the absence (n = 6) and presence of linopirdine (n = 6). Recording duration at each frequency was 20 s. **C** Comparison between averaged AP success rates at NRs at 500 Hz stimulation (n = 6). **D** Time course of AP success rate at NRs during a train of 500 Hz stimulation for 20 s. Black circles, control (n = 6); red circles, in the presence of linopirdine (n = 6). Time bin, 0.2 s. **E** Area under the curve of D for the control (open bar, n = 6) and the presence of linopirdine (red bar, n = 6). **F** Similar to (**B**) except retigabine was tested (n = 5). **G** Similar to C except retigabine was tested (n = 5). Data represent mean ± SEM, ns, not significantly different, ***p < 0.001, paired Student’s t-Test

We then investigated the potential role of KCNQ2 channels in regulating AP firing frequency at NRs. In this set of experiments, trains of stimuli at frequencies of 1, 10, 100, 200, 500, and 1000 Hz were applied to a distal end of nerves for a duration of 20 s at each stimulation frequency. Figure 7A shows sample traces of nodal APs elicited by 500 Hz stimulation for 20 s in the absence (Fig. 7A top) and presence of 100 μM linopirdine (Fig. 7A bottom). Plot of AP success rate *vs* stimulation frequency shows that AP success rate was 100% at stimulation frequency up to 100 Hz but reduced at stimulation frequency of 200 Hz or higher stimulation frequencies in control (Fig. 7B). However, AP success rate at high frequencies was increased in the presence of linopirdine (Fig. 7A–C). For example, at the stimulation frequency of 500 Hz, AP success rate was 32.5 ± 7.7% (n = 6) in the control, and significantly increased to 43.1 ± 9.1% (n = 6) in the presence of linopirdine (Fig. 7C, p < 0.001, t = 7.3). In Fig. 7D, AP success rate at 500 Hz stimulation is plotted for a prolonged period of 20 s. AP success rate in the control group gradually reduced over time and reaching steady state level after 12 s (Fig. 7D). In contrast, AP success rate was reduced to a lesser degree in the presence of linopirdine (Fig. 7D). We used the area under the curve (AUC) in Fig. 7D to compare AP success rate at 500 Hz stimulation, which shows a significantly higher AUC following the application of linopirdine (p < 0.001, t = 7.3, Fig. 7E). We also examined whether retigabine may affect AP success rate. Tested at stimulation frequency of 1, 10, 100, 200, 500, and 1000 Hz, there was no significant difference in AP success rate between control and following the application of 10 μM retigabine (n = 5, Fig. 7F, G).

## Discussion

The KCNQ2 channel has been shown to be an important regulator of membrane excitability in a variety of central and peripheral neurons [7]. In the present study we have explored regulatory functions of KCNQ2 channels at NRs of lumbar spinal ventral nerves by using whole-cell patch-clamp recording technique. We demonstrate that functional KCNQ2 channels are present at NRs of lumbar spinal ventral nerves and that these channels play a role in regulating intrinsic electrophysiological properties and saltatory conduction at NRs of the motor nerves. To our knowledge this is the first whole-cell patch-clamp recording study on roles of KCNQ2 channels at NRs of myelinated nerves. The presence of functional KCNQ2 channels at NRs of lumbar spinal ventral nerves is evidenced by our findings that non-inactivating outward currents at NRs are partially inhibited by linopirdine and potentiated by retigabine. Furthermore, we show that KCNQ2-ir is clustered at NRs of lumbar spinal ventral nerves. Since KCNQ subtype(s) such as KCNQ3 are not found to be expressed in large diameter axons of PNS nerves [10, 25], homomeric KCNQ2 is most likely the functional KCNQ channel at NRs of lumbar spinal ventral nerves.

KCNQ2 channels significantly contribute to functional voltage-gated K^+^ channels at NRs of lumbar spinal ventral nerves. The presence of voltage-gated K^+^ channels at NRs of lumbar spinal ventral nerves have recently been demonstrated by voltage-gated K^+^ channel blockers TEA and 4AP plus Cs^+^ which partially inhibit non-inactivating outward currents at the NRs [28]. A previous immunohistochemical study shows that Kv3.1b channels are also expressed at NRs of CNS nerves [9]. However, it is currently unknown whether voltage-gated K^+^ channels other than KCNQ2 may be expressed at NRs of PNS nerves [9, 10, 25]. It should be noted that most part of non-inactivating outward currents at NRs are mediated by K2P channels in myelinated nerves of PNS [5, 15, 28]. In the present study, retigabine only slightly potentiated non-inactivating outward currents at NRs, which may be partially due to the overwhelming K2P channels at NRs of these nerves.

The present study demonstrates a key role of KCNQ2 channels in regulating intrinsic electrophysiological properties at NRs of lumbar spinal ventral nerves. This is evidenced by the effects of linopirdine on membrane and action potential properties at NRs. For example, linopirdine significantly depolarizes resting membrane potentials at NRs, indicating that KCNQ2 channel activity contributes to resting membrane potential at NRs. Consistently, it has been previously suggested that a fraction of about 35% of KCNQ channels at NRs is activated at the resting membrane potential of − 77 mV [24]. The role of KCNQ2 channels in resting membrane potential at NRs is similar to that of K2P channels which maintain very negative resting membrane potential at NRs. Very negative resting potential at NRs [15] reduces steady-state inactivation of voltage-gated Na^+^ channels to ensure the availability of voltage-gated Na^+^ channels for action potential upstroke [3, 17]. We show that linopirdine significantly increases AP width, suggesting that KCNQ2 channels may participate in AP repolarization at NRs of lumbar spinal ventral nerves. In contrast, in trigeminal Aβ-afferent nerves, voltage-gated K^+^ channels did not appear to play a significant role in AP repolarization since voltage-gated K^+^ channel blockers did not significantly affect AP width at NRs of the somatosensory nerves [15]. We show that linopirdine significantly increases input resistance at NRs, suggesting that KCNQ2 channels are partially active to contribute to basal membrane conductance near resting membrane potential. This is consistent with KCNQ2 channels being a low-threshold voltage-gated K^+^ channels [24]. The increase of input resistance at NRs may account for the significant decrease of AP rheobase by linopirdine in the present study. The effects of linopirdine on the intrinsic electrophysiological properties at NRs of lumbar spinal ventral nerves are generally consistent with our recent study using classical voltage-gated K^+^ blockers including TEA and 4-AP plus Cs^+^ [28]. In contrast to linopirdine, we show that retigabine reduces input resistance and increase AP rheobase, a results further support the role of KCNQ2 in regulating intrinsic properties at NRs of lumbar spinal ventral nerves.

KCNQ2 channels play a role in regulating excitability at NRs of lumbar spinal ventral nerves, as is evidenced by the effects of linopirdine on AP firing patterns. We show that depolarizing current steps usually elicit single AP firing at NRs in the absence of linopirdine, but evoked multiple AP firing in the presence of linopirdine. Consistently, in isolated single nodes from sciatic nerve axons, direct current injection evokes more action potentials in the present of KCNQ channel blocker XE991 [25]. These results suggest that KCNQ2 channels normally exert a role to limit multiple AP firing at NRs of lumbar spinal ventral nerves. This function of KCNQ2 channels at NRs may be important for keeping high fidelity conduction of neural information.

We demonstrate that KCNQ2 channels regulate saltatory conduction at NRs of lumbar spinal ventral nerves as is evidenced by the reduction of AP conduction velocity by retigabine and the increase of AP success rate by linopirdine. Consistently, retigabine slows axonal conduction in isolated rat peripheral nerves and the effect is reversed by application of linopirdine or TEA [10]; KCNQ channel blocker XE991 decreases spike-frequency adaptation (i.e. increase of AP success rates) in rat motor axons [25]. Linopirdine shows no significant effect on AP conduction velocity in the present study. Consistently, voltage-gated K^+^ channel blockers TEA and 4-AP plus Cs^+^ also had no significant effect on AP conduction velocity in our recent study [28]. Interestingly, while linopirdine increases AP success rates at high frequency stimulation in the present study, voltage-gated K^+^ channel blockers TEA and 4-AP plus Cs^+^ produced an opposite effect in our recent study [28]. The discrepancy may suggest that in addition to KCNQ2 channels, other ion channels sensitive to TEA and 4-AP plus Cs^+^ may be present at or near NRs to participate in regulation of saltatory conduction.

KCNQ2 channel is an important target of neurotransmitters acting through a variety of Gq-coupled metabotropic receptors to regulate neuronal excitability [7]. It would be interesting to know in future whether KCNQ2 channels at NRs may also be modulated by neurotransmitters and thereby altering intrinsic electrophysiological properties and saltatory conduction in motor nerves. KCNQ2 channels at NRs of motor nerve fibers may also be explored as therapeutic targets to modulate saltatory conductions pharmacologically with inhibitors and activators of KCNQ2 channels. It would be interesting to investigate whether KCNQ2 activators and inhibitors may be used to treat myokymia, neuromyotonia, and other neuromuscular disorders.

## Data Availability

All data generated or analysed during this study are available from corresponding author on reasonable request.
